# Validation of Immune Cell Modules in Multicellular Transcriptomic Data

**DOI:** 10.1371/journal.pone.0169271

**Published:** 2017-01-03

**Authors:** Gabriele Pollara, Matthew J. Murray, James M. Heather, Rachel Byng-Maddick, Naomi Guppy, Matthew Ellis, Carolin T. Turner, Benjamin M. Chain, Mahdad Noursadeghi

**Affiliations:** 1 Division of Infection & Immunity, University College London, London, United Kingdom; 2 UCL Advanced Diagnostics, University College London, London, United Kingdom; 3 Department of Neurodegenerative Disease, UCL Institute of Neurology, University College London, London, United Kingdom; Queen's University Belfast, UNITED KINGDOM

## Abstract

Numerous gene signatures, or modules have been described to evaluate the immune cell composition in transcriptomes of multicellular tissue samples. However, significant diversity in module gene content for specific cell types is associated with heterogeneity in their performance. In order to rank modules that best reflect their purported association, we have generated the modular discrimination index (MDI) score that assesses expression of each module in the target cell type relative to other cells. We demonstrate that MDI scores predict modules that best reflect independently validated differences in cellular composition, and correlate with the covariance between cell numbers and module expression in human blood and tissue samples. Our analyses demonstrate that MDI scores provide an ordinal summary statistic that reliably ranks the accuracy of gene expression modules for deconvolution of cell type abundance in transcriptional data.

## Introduction

Transcriptomic analysis from tissue samples is now a common approach for in vivo systems level assessments of multicellular biological processes. This has allowed identification of enriched functional pathways using well-established bioinformatic tools [1–3], but deconvoluting the data to decipher the relative cellular composition of tissue samples from transcriptional profiles remains a substantial challenge [4]. To do so, multiparameter transcriptional gene signatures or modules have been proposed to reflect different cell types, and can be represented by a single summary measurement. This approach reduces noise from individual constituent genes whilst allowing comparative assessments between samples [5,6]. Modules have been used successfully to reveal relative enrichment of immune cell types in blood transcriptomic datasets [4,7–9], as well as confirming cellular infiltration in tissue inflammation, such as the site of the tuberculin skin test (TST) [10,11].

Diverse methods have been used to derive cell-type specific transcriptional modules. These include manually curated lists of genes [10], identification of genes which are differentially expressed between purified cell types [12,13], semi-supervised approaches that identify genes co-correlated with putative cell markers [11] and entirely unsupervised network analyses of purified cell types [14] or peripheral blood samples [4]. Consequently, there are now many published modules that share similar cellular annotations [4,12–14] but may vary in their constituent genes. However, there has been no systematic assessment of the comparative sensitivity and specificity with which they reflect their purported cellular associations. We tested the hypothesis that substantial discordance exists between the performance of different transcriptional modules representing individual immune cell subsets. In order to identify the transcriptional immune cell modules that reflect the cellular composition most accurately, we propose a modular discrimination index (MDI) that ranks the relationship of each module with cell numbers from human blood and tissue specimens and thereby identifies the best performing cell type specific modules.

## Results

### Derivation and comparison of cell-type specific modules

Numerous immune cell transcriptional modules have been derived by identification of co-correlated expression networks from purified cell types or human peripheral blood transcriptional datasets [14,4]. Modules can also be derived from genes differentially expressed between a cell of interest and all other cells within any one transcriptional dataset [13,15]. In addition, we derived further modules using two alternative strategies. First, by identifying transcripts with >2-fold increased expression in the cell of interest compared to all other cells common across multiple datasets from purified cells. Second, by using validated cell type specific ‘markers’ as baits to identify co-correlated genes and assemble modules with the genes that were common to all the co-correlated lists in multiple data sets [11]. These modules are listed in S3 Table.

**Fig 1:**
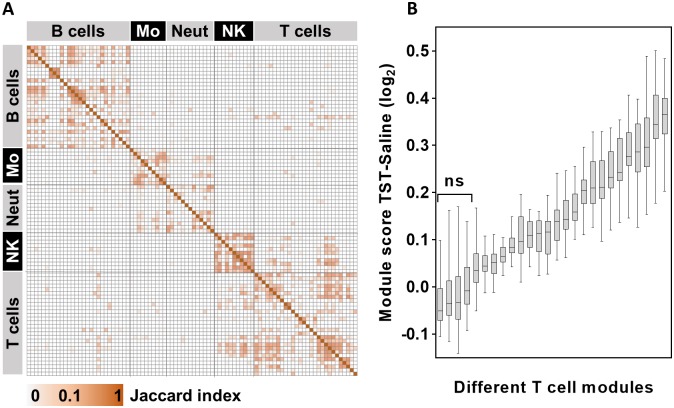
Variability in cell-type specific module content and expression. **(A)** Heat map of Jaccard index for multiple pairwise comparisons between a wide range of cell-type specific transcriptional modules, reflecting the proportion of shared genes. Mo = monocytes, Neut = neutrophils, NK = NK cells. **(B)** Geometric mean expression of all available T cell associated modules within genome wide transcriptional data from skin biopsies at the site of TST compared to the site of control saline injection. Box and whisker plots show median, interquartile range and full range of data from 16 TST biopsy sites. NS = no significant difference in data from the site of TSTs compared data from the site of saline injection.

A consequence of the multiple strategies to generate cell-type associated transcriptional modules is that a large number of independently derived modules have now been ascribed similar or overlapping annotations. We compared the constituent genes across all of the published cell-associated modules, using the Jaccard index to quantify the proportion of shared genes in all pairwise analyses. Different modules associated with the same cell type broadly revealed a greater degree of sharing, but there was also substantial discordance between modules associated with the same cell, and in some cases, substantial sharing amongst modules associated with different cell types (Fig 1A). Given the evident discordance in modules associated with a particular cell type, we tested the hypothesis that different modules would indicate significant variation in the abundance of the target cell within any given multicellular transcriptome. We compared the expression of 26 modules that have been associated with T cells (S3 Table) in genome-wide transcriptional data from the site of a tuberculin skin test (TST), representing a classical model of inflammation in which enrichment of T cells is well established [10,16,17]. Most T cell modules showed increased relative expression in data from TST samples compared to that of control saline injections, but there was striking heterogeneity in the relative expression of different modules, some of which showed no significant enrichment (Fig 1B).

**Fig 2:**
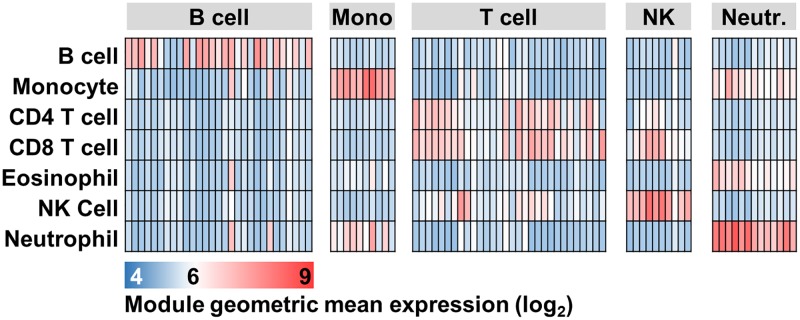
Cell-type specific module expression across multiple cell types. Heat map of module geometric mean expression for multiple cell-type specific modules (columns) within genome wide transcriptomes from selected immune blood cells (rows). Module expression calculated from E-GEOD-28491 dataset. Mono = monocytes, NK = NK cells, Neutr. = neutrophils.

In view of the heterogeneity in the performance of T cell modules, we sought to evaluate the sensitivity and specificity of a broader range of cell associated transcriptional modules, with the aim of ranking how closely each module represents the associated or target cell type. We compared the module expression levels across transcriptomic data from a variety of purified immune cell types. Although we observed higher module expression in the target cell type, this analysis revealed marked variation in signal intensities for target cells relative to others, reflecting substantial variation in both sensitivity and specificity across the range of purported cell type associated modules (Fig 2).

In order to quantify this variation we propose a molecular discriminating index (MDI) as a normalised measure of the expression of a module amongst target cells compared to all other cell types in any given dataset (S4 Table). In order to ensure the generalisability of this index, we calculated the MDI for each module in multiple datasets with sufficient breadth of purified cell types such that the cell type of interest was compared to at least four other cell types.

**Fig 3:**
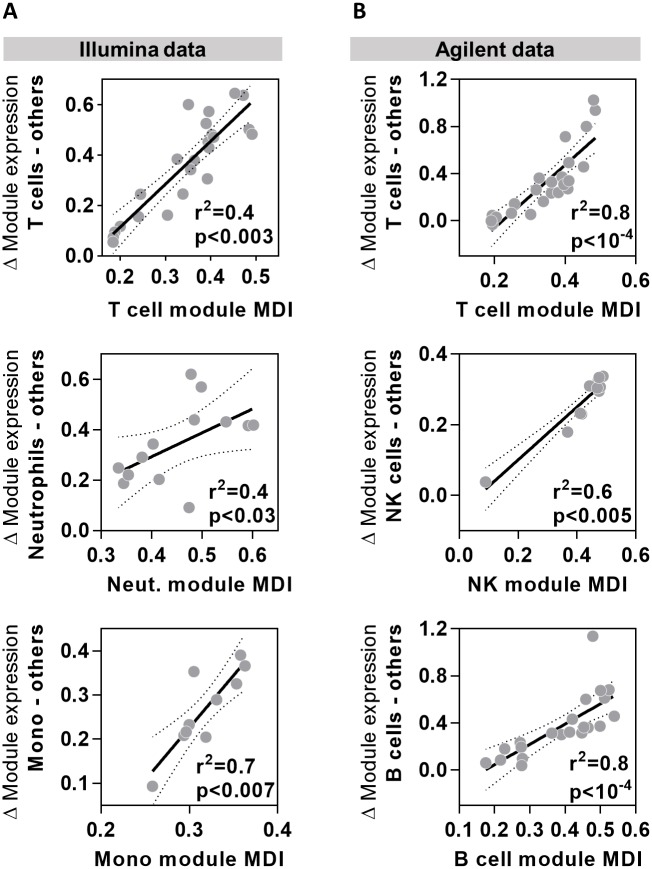
Cell type module MDI scores correlate closely with difference in gene expression between target and non-target cells. Relationship between the module MDI and relative difference in geometric mean expression of selected cell-type specific modules in data from target cell types compared to all other cell types derived from **(A)** Illumina (E-GEOD-19443) and **(B)** Agilent microarray (E-GEOD-6887) data. Data points represent data derived from individual modules, giving regression lines with 95% confidence limits, Spearman rank correlation coefficients (r^2^) and p values.

Such publicly available data were only available on the Affymetrix microarray platform, but we found that the MDI derived from Affymetrix data strongly correlated with the relative difference in module expression between target and non-target cells in data from both Illumina and Agilent microarrays (Fig 3).

### MDI score validation: Correlation with cell enrichment

**Fig 4:**
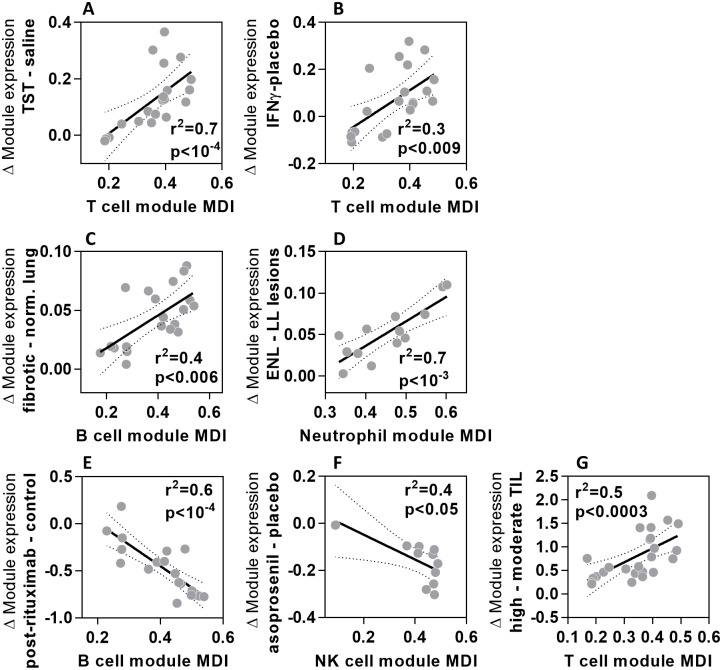
MDI scores identify modules that best reflect changes in cell number in tissues. Relationship between the module MDI and relative difference in geometric mean expression of cell-type specific modules for **(A)** T cells at the site of TST compared to saline injections; **(B)** T cells in psoriatic skin injected with IFNγ compared to saline; **(C)** B cells in pulmonary fibrosis tissue compared to normal lung; **(D)** neutrophils in erythema nodosum leprosum (ENL) skin lesions compared to lepromatous leprosy (LL) skin lesions; **(E)** B cells in blood samples from patients before and after receiving rituximab therapy; **(F)** NK cells in the uteri of women treated with 25mg asoprisinil compared to placebo; **(G)** T cells in head and neck tumours with high compared to moderate levels of tumour infiltrating lymphocytes (TIL). Data points represent data derived from individual modules, giving regression lines with 95% confidence limits, Spearman rank correlation coefficients (r^2^) and p values.

Next we sought to confirm that the MDI scores derived from peripheral blood cells correctly ranked the modules which best correlated with specific cellular enrichment within tissues identified by immunohistochemistry. In order to address this question, we surmised that modules with the highest MDI score would provide the most sensitive measure of cell-type specific enrichment. Accordingly, we found that the relative expression of each T cell module at the site of TST, compared to saline injection, correlated significantly with the MDI for T cell modules (Fig 4A). Likewise, a significant correlation was seen between MDI and relative enrichment of T cell modules in data from skin samples in which T cell infiltration was induced by injections of interferon gamma (IFNγ) (Fig 4B) [18]. B cell module MDI correlated significantly with differences in B cell module expression between tissue samples from idiopathic pulmonary fibrosis that showed B cell enrichment compared to healthy lung (Fig 4C) [19]. In erythema nodosum leprosum lesions characterised by neutrophil infiltration that is not evident in lepromatous leprosy lesions [20], enrichment of neutrophils was closely correlated to the MDI score of neutrophil modules (Fig 4D). B cell depletion after rituximab treatment [21] and NK cell depletion in the uteri of women treated with the progesterone receptor modulator asoprisinil [22] were best identified by B and NK cell modules with the greatest MDI scores (Fig 4E & 4F). These findings were observed in both Agilent and Affymetrix microarray data, but in order to extend our MDI validation further, we identified RNA-Seq expression data from human head and neck cancer samples where tumour infiltrating lymphocyte (TIL) numbers were classified as high or moderate [23]. The differences in T cell module expression between high and moderate TIL tumours closely correlated with T cell module MDI score (Fig 4G). These analyses validated the use of MDI to rank the modules which best reflect variation in the abundance of specific cell types within tissue using transcriptomic data from diverse microarray platforms and RNA-Seq.

### MDI score validation: Covariance with cell numbers

We sought to further validate module MDI ranking by comparing this statistic to other methods for quantitation of cell frequency in various human tissues. We used the covariance between cell frequency and module gene expression as a measure of the sensitivity of each module to detect the abundance of its associated cell type. We then hypothesised that this covariance would be closely correlated with module MDI scores independently derived from purified cell data. Modules which showed the highest covariance with target cell numbers would therefore be predicted to have the highest MDI.

**Fig 5:**
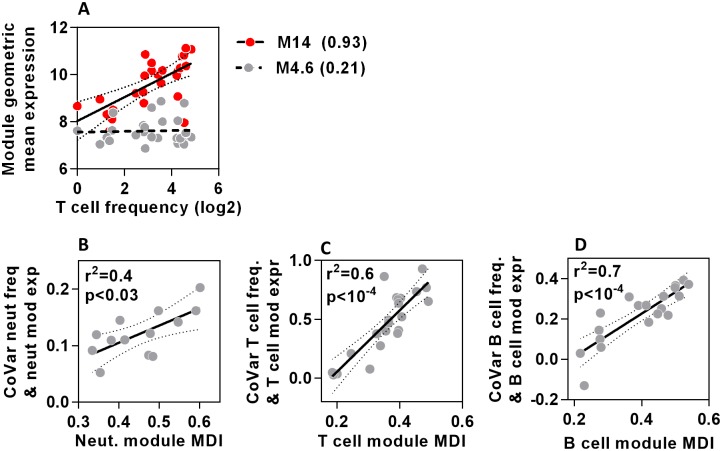
MDI scores predict strength of covariance between module gene expression and cell frequency in blood. **(A)** Relationship between geometric mean expression of two T cell modules which show highest (M14) and lowest (M4.6) covariance (shown in brackets) with T cell frequency in peripheral blood. Each data point represents measurements derived from a TST in separate individuals. Regression lines and 95% confidence limits are shown for each module. **(B-D)** The relationship between covariance of neutrophil, T cell or B cell frequencies and cell-type specific module expression, with the MDI for each module. Data points represent data derived from individual modules, giving regression lines with 95% confidence limits, Spearman rank correlation coefficients (r^2^) and p values.

Peripheral blood transcriptomes from samples with known differential blood cell counts provided the opportunity to compare variation in cell type module expression with that of directly measured cell frequency. Blood transcriptomes of patients presenting to hospital with febrile illnesses [24] confirmed that different modules associated with the same cell type show striking heterogeneity in their covariance with actual cell counts (Fig 5A). As hypothesised, MDI scores for neutrophils, T cell and B cell modules each showed excellent correlation with the covariance between module expression and cell frequency (Fig 5B–5D). Importantly, this analysis spanned cellular frequencies ranging from 0.1-94% of the total leucocyte fraction, suggesting that the ranking of modules by MDI score remains valid even at very low cell frequencies.

**Fig 6:**
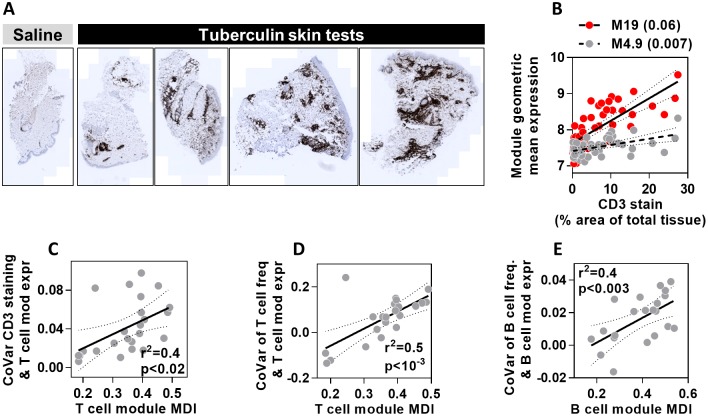
MDI scores predict covariance between module gene expression and cell frequency in skin and lymph nodes. **(A)** Representative images of CD3 immunohistochemical staining in skin biopsies at the site of saline injection or tuberculin skin tests. **(B)** Relationship between geometric mean expression of two T cell modules which show highest (M19) and lowest (M4.9) covariance (shown in brackets) with quantitation of CD3 immunostaining. Each data point represents measurements derived from a TST in separate individuals. Regression lines and 95% confidence limits are shown for each module. **(C)** The relationship between covariance of CD3 immunostaining in TST biopsies and T cell module expression, with the MDI for all T cell modules. **(D-E)** The relationship between covariance of T or B cell frequency and cell-type specific module expression, with MDI for each module. Data points represent data derived from individual modules, giving regression lines with 95% confidence limits, Spearman rank correlation coefficients (r^2^) and p values.

We extended our cell frequency covariance analysis in two ways. First, we assessed an additional TST dataset for which parallel immunohistochemistry staining for the T cell marker CD3 was performed, showing a range of CD3+ T cell infiltration in different individuals (Fig 6A). The covariance between CD3 staining in the skin and the expression of different T cell modules was variable (Fig 6B). This covariance showed strong correlation with T cell module MDI scores (Fig 6C), confirming that modules with the highest MDI provided most sensitive measurement of differences in T cell abundance. In addition, we used a transcriptomic dataset combined with flow cytometric analysis of disaggregated lymph node (LN) biopsies from patients with follicular lymphoma [21]. In this example also, module MDI scores correlated closely with the covariance between relative proportions of T and B cells and the expression of each cell-type specific module (Fig 6D & 6E). We conclude that the MDI score successfully ranks the cell type modules that best describe the relative frequencies of different immune cells within tissues.

## Discussion

Multiple gene modules, generated by different approaches, have been described for many different cell types which seek to provide a discriminatory transcriptional signature [4,12–14]. Crucially, the process of predicting the overall cellular association of modules from its constituent genes is often manual and dependent on investigators’ a priori knowledge, or interpretation of the published literature. The analysis presented in this paper demonstrates that different modules purported to represent the same cell type vary significantly in their specificity, and consequently provide different estimates for cell type enrichment in tissues.

We quantified module performance by relating it to module specificity as measured by a meta-analysis of module expression across a large set of purified immune cell transcriptomes. This specificity was then mapped to an ordinal scoring system which we call MDI. We demonstrate that MDI scores accurately predict relative module enrichment in tissues and, where available, predict module expression covariance with cell numbers in both blood and tissue. A strength of this scoring framework is that it provides an independent hierarchy of module accuracy applicable across different tissue settings and across different sources of transcriptional data, including several microarray platforms and RNA sequencing. MDI scores therefore provide a good predictor of module performance in cell type quantification, without requiring extensive in silico or in vitro validation of module performance.

We focused on generating MDI scores for immune cell modules as changes in cell numbers are common biological questions and many datasets of pure cells were available for scoring purposes. Importantly, we showed that the MDI score correctly ranked the sensitivity and specificity of the cell type specific modules within clinical samples representing a diverse array of pathologies or immunological responses. We interpret this to mean that the best performing modules represent core transcriptional programs independent of differential cellular activation states. Although we and others have described cytokine and innate immune stimulus specific gene expression modules to represent functional biological activity beyond cellular composition [11,25,26], comprehensive data from which to derive transcriptional modules representing cell-type specific activation states are not presently available, and may well require multiparametric flow cytometry to resolve accurately. Likewise, transcriptional data to discriminate between closely related cell subsets, such as CD4 and CD8 naïve or memory T cells are the focus for future work.

The meta-analysis performed in this study supports the use of transcriptional modules to extract biological information from tissues as well as blood. Modules have been mostly used to describe relative cell and pathway enrichment in whole blood or peripheral blood mononuclear cells (PBMC) [8,9]. We previously identified transcriptional modules which could decipher cell and innate immune response enrichment from human skin [11]. In the current manuscript we extend these observations further, demonstrating their applicability across a range of human tissue types. Although some cells undoubtedly induce context specific transcriptional programmes, we demonstrate that cells retain some tissue-agnostic transcriptional profiles, observing that B cell modules with the greatest specificity (i.e. MDI score) accurately reflect B cell numbers in lungs, blood and lymph nodes. Equally, the most specific T cell modules reflect T cell numbers in blood, head and neck tumours, skin and lymph nodes. Notably, we also show for the first time that cell biology in tissues is faithfully reflected by modules derived purely from unsupervised network analysis of whole blood transcriptomes [4].

The MDI scoring framework also provided insight into the merits of different module generating methodologies. Whilst no single approach consistently provided the best modules, MDI scores highlight that unsupervised network analyses generate highly heterogeneous modules, some possessing no specificity for their annotated cell type. Secondly, where modules are generated in a supervised manner by incorporating genes which show differential expression, limiting a module’s constituent genes to those identified in multiple datasets almost always improves performance compared to using analysis of individual datasets. Finally, in settings where dataset availability may be limited or novel cell subtypes are being investigated [27], a semi-supervised approach to module generation using genes co-correlated to marker bait genes appears to be a viable alternative approach to generate highly specific modules [11].

In conclusion, we have generated and validated in multiple settings a novel, cross-platform framework for ranking the quality of published transcriptional modules. We propose that the MDI scoring approach becomes a new standard to assess the fidelity of modules’ annotations in order to make accurate comparative assessments in transcriptional analyses.

## Methods

### Datasets

All datasets used are described in S1 Table. Data matrices were obtained from processed data series downloaded at the Array Express repository (https://www.ebi.ac.uk/arrayexpress/). Probe identifiers were converted to gene symbols using platform annotations provided with each dataset. In circumstances where downloaded datasets were not log2 transformed, this was performed on the entire processed data matrix. Datasets were alphabetically sorted by gene symbol and duplicate genes were removed after the first one identified using Microsoft Excel duplicate remover function.

### Scripts

All scripts were written in R v3.2.2 and RStudio v0.98, and are available to download on GitHub (https://github.com/MJMurray1/MDIScoring). A list of functions used in the manuscript is available in S2 Table.

### Module derivation and generation

All module names, annotations and gene contents used in the analyses are available in S3 Table. In addition to published cell-associated modules, we derived further modules using two alternative approaches. First by identifying overlapping module content across multiple datasets composed of purified cell types (accession numbers E-GEOD-22886, E-GEOD-28490, E-GEOD-28491, E-GEOD-24759 and E-GEOD-50008). For every gene, the expression difference between the cell type of interest and all other cell types in the dataset was computed. Genes that were two-fold or greater upregulated in the target cell compared to at least 90% of the other cell types in that dataset were selected. This process was then repeated for all datasets, and genes identified in at least two datasets were then selected to comprise the final module.

Secondly co-correlation modules were generated using cell specific marker genes derived from the published literature as previously described [11]. The Pearson correlation coefficient of each marker relative to all other genes was derived from a dataset comprising a wide variety of purified cells (E-GEOD-22886). This approach yielded a ranked list of genes correlated to each marker, and the cell module was then constructed from the overlap of the top 1% of correlated genes in each of the lists.

### Assessing module content overlap

Pairwise assessment of module similarity was made using the Jaccard similarity index, which is defined as the intersection divided by the union of two sets (S2 Table). Content for both assessments was derived from HGNC gene symbols in S3 Table.

### Calculating MDI scores

The overall gene expression of any module was determined from the geometric mean expression of all the constituent genes. MDI scores were calculated from datasets containing a wide breadth of purified cell types in which the cell type of interest made up <20% of all cells (range 4.7% - 16.7%—S1 Table). Within each data matrix, we identified the samples that best represented each cell type (e.g. when calculating MDI scores for B cells, the prototypic condition across the datasets tested was “B cells from PBMC”, “B cells” or “naïve B cells”). All other non-target cell types then were assigned “Other” status.

Modules for which the mean expression across multiple datasets in the target cell was lower than any other cell type were excluded from further analysis. Modules’ MDI scores were then calculated from the cross-dataset average of relative geometric mean expression difference between the target cell and all other cell types according to the equation:

$$MDI_{mod}=\frac{1}{n}\sum_{i=1}^{n}\left(\frac{X-Y}{X}\right)$$

$$MDI_{mod}=MDI\;score\;for\;module\;annotated\;as\;target\;cell$$

X=Module geometric mean for target cells in dataset

Y=Module geometric mean for non−target cells in dataset

n=number of datasets used to derive MDI score

Where a dataset specified the differentiation or activation state of a target cell, these conditions were averaged prior to MDI scoring, in order to focus on modules’ core performance to reflect a particular cell type (e.g. gene expression of resting and activated T cells was averaged when generating MDI scores for T cells overall). All other comparator conditions in that dataset were unaltered for MDI score generation.

### Skin biopsy collection and transcriptomic analysis

Skin biopsy samples were collected as part of a research study approved by UK National Research Ethics Service (reference no: 11/LO/1863). Written informed consent was obtained from all participants included in the study.

Healthy volunteers with positive IFNγ release assays using QuantiFERON-TB Gold tests (Qiagen) received 0.1 ml intradermal injection of two units tuberculin (Serum Statens Institute) or saline injections in the volar aspect of the left forearm. At 72 hours, skin biopsies for transcriptomic and histological analysis were collected and stored as previously described [11]. RNA was extracted from skin, processed by Agilent microarrays and analysed according to an established pipeline that we have previously described [11,28].

### Histology and immunohistochemistry of skin biopsy specimens

Punch skin biopsies for histological analysis were snap frozen in OCT Compound (Tissue-Tek). Frozen sections were carefully thawed and fixed in 4% neutral buffered formalin, then embedded in paraffin wax (Sakura). 10 μm sections were cut and stained using the Leica Bond III automated immunostaining platform, with the Leica Bond Polymer Refine detection kit (Leica DS9800and a DAB chromogen. More specifically, anti-human antibodies against CD3 (clone LN10) (Leica NCL-L-CD3-565) were used. Whole slide images of the histology sections were acquired with an Axio-Scan microscope using Zen 2 core software at 20x magnification and are presented without any subsequent processing. Digital image analysis was performed using Definiens AG (Munich) Tissue Studio 4.3. Tissue detection automatically identified all the tissue within each image, then a machine learning method was used to separate the sample from background and non-tissue regions, and segment the sample into dermis and epidermis; manual correction was used to ensure valid separation of these regions of interest (ROI). A fixed threshold was then applied to each ROI to identify the chromogen positive areas (μm^2^), which is represented as a percentage of the total tissue/ROI area.

### Statistical analysis

Spearman Rank correlations and linear regression models were calculated in Graphpad Prism, and covariance analyses were performed in Microsoft Excel.

## Supporting Information

S1 Table Table of accession numbers for all microarray data used in this manuscript. (XLSX)

S2 Table Table of R functions utilised for all data analyses in this manuscript. (XLSX)

S3 Table Module names, descriptions, source and contents. (XLSX)

S4 Table Table of immune cell module MDI scores. (XLSX)
